# The Functional Status of Patients with AIDS Attending Antiretroviral Treatment Center

**DOI:** 10.4103/0973-1075.53513

**Published:** 2009

**Authors:** TJ Thejus, MC Jeeja, T Jayakrishnan

**Affiliations:** All India Institute of Medical Sciences, Ansari Nagar, New Delhi, India; 1 Department of Pharmacology, Medical College, Kozhikode, Kerala, India; 2 Department of Community Medicine, Medical College, Kozhikode, Kerala, India

**Keywords:** AIDS patients, Anti-retroviral treatment, Disease staging, Functional status, Palliative care

## Abstract

**Aims:**

To assess the functional status of patients with Acquired immunodeficiency syndrome (AIDS) registered in the Anti-Retroviral Treatment (ART) center.

**Materials and Methods:**

Design: Descriptive study. Study setting: ART center in Calicut Medical College, Kerala, India. Subjects: Cohorts of AIDS patients attending the ART center during the year 2007. Data collection: Done prospectively from the secondary data available from the center. Outcome measures: The demographic, morbidity, functional status and laboratory parameters were collected. Data processing was done using Excel datasheet and analysis were done using Epi info 2003.

**Results:**

One hundred and ninety-five patients received care during this period; 69% were males. The mean age was 38±9 years; 80% of them were married and in 50% of their spouses also tested positive for HIV. The mean CD4 count was 127 cells/microliter. The majority (90%) were categorized as WHO Stage 3 or 4 of HIV. Only 52% of them were able to perform their usual work in or outside their house; the rest were not able to lead an economically productive life. Thirty-six per cent were only able to perform activities of daily living; 12% were bedridden. The functional status of the patients positively correlated with WHO disease stage (*P* = < 0-0001), and CD4 count and hemoglobin levels negatively correlated with staging (*P* = <0.001). 62% are having any of the opportunistic infections.

**Conclusion:**

Fifty per cent of the AIDS patients are disabled and need support and care. As AIDS is a growing problem, community-based palliative care for AIDS patients should be strengthened in India.

## INTRODUCTION

According to UNAIDS and the World Health Organization (WHO) there are 33 million people living with HIV/AIDS around the world, of which 90% are in developing countries. During the year 2007, 2.7 million (95% CI 2.2 – 3.2 M) people were newly infected and 2.0 million (95% CI 1.8 – 2.3 M) died due to AIDS.[1] As per the latest estimates the people living with HIV/ AIDS in India are 2.31 million (95% CI 1.8 – 2.8M). The adult prevalence is 0.34% (95% CI 0.25 – 0.43), the male prevalence being 0.4% and female 0.27%.[2] Kerala was considered as a low prevalence vulnerable state as with spectrum result analysis the prevalence was reported to be 0.26%.[2] During the year 2006 the number of people living with HIV in Kerala were estimated to be 1,00,000 with 838 newly reported cases and 175 deaths.[3]

AIDS is a disease with a prolonged course finally leading to death in which effective cure is not yet available. Now the anti-retroviral treatment (ART) which was started in India as a government program during 2004 is prolonging the life of AIDS patients. Since more young persons were affected with HIV they live with various functional disabilities which need lifelong continuous care.

The stated Goal of the National AIDS Control Program III (NACP) of the Government of India is to halt and reverse the epidemic of HIV within five years. The strategies for achieving the goals are scaling up of interventions by providing ART to three lakh patients through establishing 250 ART centers throughout the country by 2012,[4] thus increasing proportions of people living with HIV and AIDS (PLHA), who receives Care, support, and treatment. Out of the total funds (11,585 crores) allocated for NACP during the 11^th^ Plan period, 16.9% were earmarked for support and treatment services (11^th^ plan, Ministry Of Health and Family Welfare, Government of India). According to national guidelines National Aids Control Organisation (NACO) will provide funds to NGOs for setting up community care centers for AIDS patients.[5]

Out of the 172 ART centers in the country six were functioning in Kerala catering centralized services in five Government medical colleges and one at the district hospital of Palakkad. The state has 101 Integrated Counseling Testing Centers (ICTC) functioning at District / Block level and six community care centers.[4,6] The Calicut district has got six ICTCs.

Under NACP III all ART centers will be linked to community care centers with outreach workers trained in home care.[4] In a country like India with poor resources, hospital care for them is not feasible and in Kerala the availability of community care centers for AIDS care is less- six in number. The establishment of community-based home care is a cost-effective solution for us. Home-based care and support system has been taken up as an integral part of NACP.[5] Kerala is the only state in the country which has formulated a palliative care policy.[7] Since hospital care is not feasible, home-based care is preferred over hospital care. The data about the functional status of AIDS patients are scarce in our country. On this background, to know the functional status of AIDS patients, a study was conducted.

## MATERIALS AND METHODS

The descriptive study was conducted at the ART center (Ushus) attached to the Kozhikode Medical College Hospital in Kerala, India (Clarification given at the end). The center cater ART services to five northern districts covering a population of about 125 million, hence the data can be representative of the state and the result can be generalized. The community care center for HIV/AIDS patients for this area was functioning in the Institute of Palliative Medicine run by the Pain and Palliative Care Society, Calicut (A WHO demonstration project), which has been functioning attached to this medical college. This center has got community based organization (CBO)-based network of palliative care services and volunteers spread out in all northern districts.

The subjects selected were cohorts of AIDS patients attending the ART center during the year 2007. The data collection was done prospectively from the secondary data available from the ART register (JMC-second author), after obtaining approval from the institutional ethical committee. From the registration point onwards each patient's data was collected periodically by subsequent follow-up visits for a one-year period from January 2007 till the end of December 2008. Demographic details like age, gender, literacy, marital status, and morbidity details like stages of HIV including opportunistic infections, HIV status of spouse and laboratory parameters like CD4 count, hemoglobin level were collected using a standardized form. The HIV staging was done as per WHO Classification of clinical staging. Stage I: Asymptomatic. Stage II: Unexplained weight loss <10%.Stage III: Unexplained severe weight loss >10%+symptoms. Stage IV: HIV wasting syndrome.[7,8] The functional status and disability was graded according to WHO criteria which has been validated and accepted globally.[8,9] The grading was as follows: A) Working: Able to perform usual work inside or outside home. B) Ambulatory: Able to perform Activity of Daily Living-ADL, Not able to work. C) Bedridden: Not able to perform ADL.[9] Following the initial visit the patient's data was updated at every subsequent visit (JMC). The study was approved by the Institutional Ethics committee -Kozhikode Medical College.

The data processing was done using Excel data sheet and analysis were done using Epi info 2003 (TTJ-first author).The discrete variables were expressed as proportions and quantitative variables were expressed as means +SD and the significance was fixed at a *P* value of <0.5.

## RESULTS AND DISCUSSION

One hundred and ninety-five patients received care from this center during this period (January 2007 to December 2008). The baseline demographic data was as follows:

The majority (69%) were males, which was the same as for the Y R Gaitonde Center for AIDS Research and Education YRG cohort data from south India.[10] In the International Epidemiological Database to Evaluate AIDS-lower income countries (IeDEA-LINC) data of 15 countries the male proportion was 54%.[11] While showing the increased incidence of cases in men it also indicates the reporting bias due to the social norms of increased service utilization and early diagnosis of male cases. The mean age was 38 + 9 years. In all the databases from the east and west the mean age of patients at the presentation for starting of care was 35-45 years.[10] In the YRG cohort the mean age was 32 years. The majority were in the economically productive age group and may be the only support to their family hence the morbidity may push their family to impoverishment. Eighty per cent of them were married and in 50% their spouses also tested positive for HIV. It can be seen that as per the national surveillance data, the majority (48%) were of productive age group.[2] Seventy per cent of the patients came from four other districts; the distances ranging from 50 to 250 km. Hence access to transport is also a major problem for them.

The CD4 count is a proxy indicator of severity of disease which corresponds to the functional status and reflects the immunocompromised state of the patients. The mean CD4 count was 127 cells micro liter [Table 1] which was lower than the YRG cohort data of 316 cells/micro liter and higher than the IeDEA ART-LINC data of 105 cells/micro liter.[8,9] But from the same data of patients from Asian countries the mean CD4 count was 83 cells/micro liters.[9] According to WHO and NACO low CD4 count below 200 cells micro liter requires treatment.

**Table 1:** Baseline characteristics (n=195)

Variable	Mean	95% CI
Age	38.18 Years	36.90 – 39.46
Weight	48.54 Kg	46.71 – 50.37
CD4 count	127.60 cells/micro liter	100.6 – 154.58
HB%	10.75 gm%	10.33 – 11.17
Literacy rate		NA
Illiteratess	3.2%	
Primary school	−33.3%	
Secondary school	−36.4%	
College	−08.7%	
Opportunistic infections		NA
Total present	−62%	
TB	−33.3%	
Candidiasis	−36%	
PCP	−8.7%	

The blood hemoglobin level is another determinant of functional status. According to the hemoglobin status the majority were anemic (mean HB −10.8 gm %) which reflect the poor nutritional status of the AIDS patients attending here [Table 1]. According to National Family Health Survey III (NFHSIII) data the prevalence of anemia among the adult population in the state was 32.8% in women and 8% in men.[12]

The severity and progress of the HIV/AIDS disease status was assessed by WHO clinical staging. Stage I: Asymptomatic. Stage II: Unexplained weight loss <10%. Stage III: Unexplained severe weight loss >10% + symptoms. Stage IV: HIV wasting syndrome.[8] The distribution of patients according to staging is as follows. Stage 1-2.1%, Stage 2-6.7%, Stage 3-48.7%, Stage 4-42.5% [Table 2]. This shows that more than 90% were in Stage 3 or 4 which is a very advanced stage of disease indicating delay in reporting to the center for initiating treatment [Table 2]. In the IeDEA ART-LINC data only 31% were in Stage 3 and 4.[11] This may be due to stigma or information asymmetry in AIDS care along with accessibility to centralized care including mobility support, which were reported earlier from the state.[13] Compared to 92% literacy status of the state population, 96.8% of the patients were literate and >45% had more than five years of schooling which point out that the delay was not due to ignorance. The clinical stages of the patients have got negative correlation with the baseline CD4 count (r = −0.345, *P* = 0.0001) and body weight (r = −0.209, *P* = 0.008)

**Table 2:** WHO clinical stages and functional status of AIDS Patients

WHO clinical staging*	Baseline %	After 6 months %
Stage I	2.1	00
Stage II	6.7	19
Stage III	48.7	49.4
Stage IV	42.5	31.6
Functional status**		
Working	52.5	39.4
Ambulatory	35.8	46.8
Bedridden	11.7%	13.8%

* Stage I: Asymptomatic. Stage II: Unexplained weight loss <10%. Stage III: Unexplained severe weight loss >10% + symptoms. Stage IV:HIV wasting syndrome

** a)Working: Able to perform usual work inside or outside home. b) Ambulatory: Able to perform Activity of Daily Living -ADL, Not able to work. c) Bedridden: Not able to perform ADL

According to the grades of disability, the functional status of the HIV patients was categorized into three grades by WHO, which was internationally validated. It is followed by NACO and used in all ART centers in India.[9] The grading was as follows-A) Working: Able to perform usual work inside or outside home. B) Ambulatory: Able to perform Activity of Daily Living -ADL, Not able to work. C) Bedridden: Not able to perform ADL.[9] At the entry point the functional status was as follows: Working-52.5%, Ambulatory −35.8%, Bedridden-11.7%. Only 52% of them were able to perform usual work in or outside their house; 36% were only able to perform their activity of daily living; 12% were bedridden [Table 2]. Forty-eight per cent could not work and needed economic support; the 12% who were bedridden needed help for daily life activity like eating, dressing, toilet and moving indoors. The functional status of patients at the entry level had a positive correlation with their disease stage (r = 0.365, *P* = 0.0001) and negative correlation with CD4 count (r = −0.198, *P* = 0.012).The functional status worsened during follow-up visits after six months and at this point only 39% were able to work [Table 2]. Since AIDS is a chronic progressive disease with a prolonged course,[9] the proportion of patients who need support increases in due course.[4,5]

Due to stigma and discrimination towards people living with HIV (PLHIV) existing in our society even family members are reluctant to take care of the AIDS patients.[2,4,5] Sometimes, the only caretaker was the spouse, also positive for AIDS. In this cohort 16% had no spouse to support them. Among them 6.5% were unmarried, 3.8% were divorced and 5.4% were widow/ers. Due to their own morbidity problem, 50% of the spouses who are HIV-positive themselves may not be able to give physical support or care to them. Compared to the general population the probability of living with disability or dying within a short time among them was also high. Since the mean age was 38 years, the majority of them had no grown-up children to take care of them.

AIDS patients are prone to various opportunistic infections. Sixty-two per cent of the studied cohort had many of the opportunistic infections associated with AIDS which worsen their disability and quality of life. At the entry level itself 33.3% were suffering from Tuberculosis (TB), 36% were affected with Candidiasis and 8.7% had pneumocystis carnie pneumonia [Table 1]. Among those who had any opportunistic infection (OI) 66% were disabled for working outside, as compared to 32% among those without any OI (*P* = <0.05). In the future multiple disease burden and recurrent infections would make their life crippled making supportive care from others a necessity.

## CONCLUSIONS AND RECOMMENDATIONS

Forty-eight per cent of the AIDS patients had disabilities [Table 2], were not able to work or were bedridden and needed supportive care from the community. This data was collected at the entry stage. After six months' follow-up the proportion of patients with disability had increased to 69% [Table 2] and may worsen during the course of the disease in the following days.

As AIDS is a growing problem in India and Kerala and ART has prolonged the life expectancy of PLHIV, we have to expect more PLHIV with functional disability in our society. Studies around the world have shown that the mortality decreased rapidly after the initiation of ART.[9] Since hospital-based care is not feasible home or community-based care is our appropriate option. Medecins Sans frontiers (MSF) programs at Malawi showed that community support improves the outcome of AIDS care.[9] In a low-resource state like ours where only 0.9% of the Gross Domestic Product (GDP) is allocated for health, community-based palliative care for AIDS patients is a real health need which should be strengthened as priority. Compared to other parts of the state this part has got tremendous (70%) coverage of community-based palliative care services like neighborhood network in palliative care (NNPC) with support of local self governments (7). We recommend that the NNPC should also focus on the issues of AIDS care in future.

### Limitations of the study

Data was collected from a tertiary care hospital from those who were attending ART center and hence got reporting bias. Large proportions of AIDS patients in the community were not able to report to the center due to some/any sort of functional disability and accessibility problems hence the chance of under-reporting was high.

The data was collected from the ART treatment register at the center and not directly from the patients.
